# Advanced Level Practice Education: UK Critical Care Pharmacists’ Opinions in 2015

**DOI:** 10.3390/pharmacy4010006

**Published:** 2016-01-22

**Authors:** Ruth E. Warin, Richard S. Bourne, Mark Borthwick, Greg Barton, Ian Bates

**Affiliations:** 1 Departments of Pharmacy and Critical Care, Sheffield Teaching Hospitals, Northern General Hospital, Herries Road, Sheffield S5 7AU, UK; Richard.bourne@sth.nhs.uk; 2 Pharmacy Department, John Radcliffe Hospital, Headley Way, Oxford OX3 9DU, UK; Mark.borthwick@ouh.nhs.uk; 3 Pharmacy Department, St Helens and Knowsley Teaching Hospitals NHS Trust, Whiston Hospital, Warrington Road, Prescot L35 5DR, UK; greg.barton@sthk.nhs.uk; 4 Department of Pharmacy, University College London, 29–39 Brunswick Square, London WC1N 1AX, UK; i.bates@ucl.ac.uk

**Keywords:** advanced practice, pharmacy, education

## Abstract

National UK standards for critical care highlight the need for clinical pharmacists to practice at an advanced level and above. The aim of this research paper was to describe the views of UK critical care pharmacists on the current provision of Advanced Level Practice (ALP) education and accreditation. It sought to identify whether there is a need for a national or regional training programme. A questionnaire was delivered electronically targeting UK critical care pharmacists. Whilst the response rate was low at 40% (166/411); the views expressed were representative of UK practitioners with the majority of responders meeting the national specifications for clinical pharmacist staffing in critical care areas. The responses highlighted work-based learning as the main resource for developing ALP and a lack of suitable training packages. The vast majority of pharmacists identified that a national or regional training programme was required for ALP. The results also identified the main barriers to undertaking ALP accreditation were lack of time, uncertainty regarding the process and its professional benefits and a lack of education and training opportunities. In conclusion, the responses clearly indicated that, for the necessary progression of critical care pharmacists to ALP, a national or regional training programme is required.

## 1. Introduction

In the UK, specialist pharmacists working in critical care have started to engage in the process of developing Advanced Level Practice (ALP). A competency framework for ALP in critical care pharmacy was developed by the United Kingdom Clinical Pharmacy Association Critical Care Group (UKCPA CCG) in 2003 [1]. This was underpinned by a syllabus of knowledge required for pharmacists practicing in critical care, and, subsequently, a credentialling process was developed to assess this [2,3]. The process from devising the competency framework to the credentialling process has provided the basis for assessment of ALP in critical care within the newly formed Royal Pharmaceutical Society (RPS) Faculty [4].

The UK national standards for critical care include recommendations on the skill level and staffing requirements for critical care pharmacists [5]. The standards emphasise the need for critical care pharmacists to be working at ALP (Band 8a) or consultant level (Band 8b+) [5]. It indicates that pharmacists working at critical care foundation level (equivalent to Faculty Advanced Stage I (MFRPSI)) should have access to colleagues at critical care advanced level (equivalent to Faculty Advanced Stage II (MFRPSII)) or consultant level (Faculty Fellow (FFRPS)) for advice and referrals [2,4,5]. This is particularly important when dealing with the highly complex patients common to critical care practice. Currently, the UK critical care workforce data identifies that approximately half of the pharmacists feel they are working at, or below critical care foundation level. Additionally, 24.8% of NHS Trusts have a critical care pharmacy service delivered at foundation level or below [6].

There are currently no UK-based formalised training programmes that meet the needs of pharmacists seeking to develop the skills for critical care ALP framework. The scenario is similar in the US, where the training capability of specialist critical care pharmacists has been recognised as a major limitation to nationwide implementation of this clinical service [7]. The American College of Clinical Pharmacy (ACCP) also recognise that education and training is essential for the development of advanced pharmacists’ roles [8].

For the UK to deliver the necessary critical care pharmacist workforce capacity at ALP and above, an education strategy linked to workforce development is required. It is therefore important to establish the educational resources that are currently being used by critical care pharmacists. This is to ensure that their clinical practice is supported as they advance through the critical care ALP framework. Their perspective on the current resources and their opinion on the need for a national educational programme specifically developed for ALP in critical care pharmacy are required.

The aim of the questionnaire was to describe the views of UK critical care pharmacists on the current provision of ALP education including the UKCPA CCG’s masterclass study days. It also sought to identify the thoughts of pharmacists on the perceived need for a national or regional training programme for ALP.

## 2. Methodology

A prototype of the questionnaire was designed and presented to the UKCPA CCG Expert Practice Development Group^1^ who commented extensively. It was redesigned and the final version (Appendix 1) was delivered electronically using Survey Monkey^®^. A link was posted on the UKCPA CCG discussion board with subsequent reminder postings.

The questionnaire was also circulated via email to critical care pharmacists within the UK to ensure those who were not members of the UKCPA were also surveyed. The questionnaire was closed in May 2015 and analysis began in June 2015. The survey was classified as a service evaluation by the Sheffield Teaching Hospitals NHS Foundation Trust Clinical Effectiveness Unit (CEU number: 6853).

Sub-group analysis was performed according to the status results for whether the pharmacists worked in a designated teaching hospital, as part of a critical care pharmacy team and also whether they had already undertaken the UKCPA credentialing or RPS faculty assessment. A Chi-square analysis (with Yates correction for continuity) was used to compare result proportions between these sub-groups, and statistical analysis was undertaken using Sigmaplot 12 software (Systat Software Inc., San Jose, CA, USA).

## 3. Results

### 3.1. Response Rate

A total of 166 critical care pharmacists completed the questionnaire out of a potential 411 practicing within the UK at the current time. Thus, we had a response rate of approximately 40%.

### 3.2. Pharmacist Demographics

There was representation from throughout the UK; 82.6% (128/155) from England, 6.5% (10/155) from Scotland, 7.1% (11/155) from Wales and 3.9% (6/155) from Northern Ireland. 50.9% (84/165) of pharmacy responders had worked in critical care for 5 years or more and 31.5% (52/165) had worked for 2–5 years. A total of 83.4% (136/163) were Band 8a and above (Table 1). Approximately three-fifths (59.8%, 98/164) worked in a designated teaching hospital and three-quarters (74.4%, 122/164) worked as part of a critical care pharmacy team.

**Table 1:** Agenda for Change (AfC) Banding of pharmacist responders.

Afc Band	Frequency, n (%)
6	1 (0.6%)
7	26 (16%)
8a	106 (65%)
8b	26 (16%)
8c	4 (2.5%)

Note: Pharmacists entry level into the NHS is AfC Band 6 with potential progression up to AfC Band 8c or d within clinical practice.

### 3.3. UKCPA Credentialing and RPS Faculty Accreditation

Out of the 166 critical care pharmacists, 21 (12.8%) had undertaken the UKCPA credentialing or RPS Faculty accreditation. Pharmacists working in a designated teaching hospital appeared more likely to have undertaken the UKCPA credentialing or RPS faculty accreditation compared to those who did not, 17.3% (17/98) compared to 6% (4/66). However, this difference in proportions did not reach statistical significance (*p* = 0.06).

**Figure 1:**
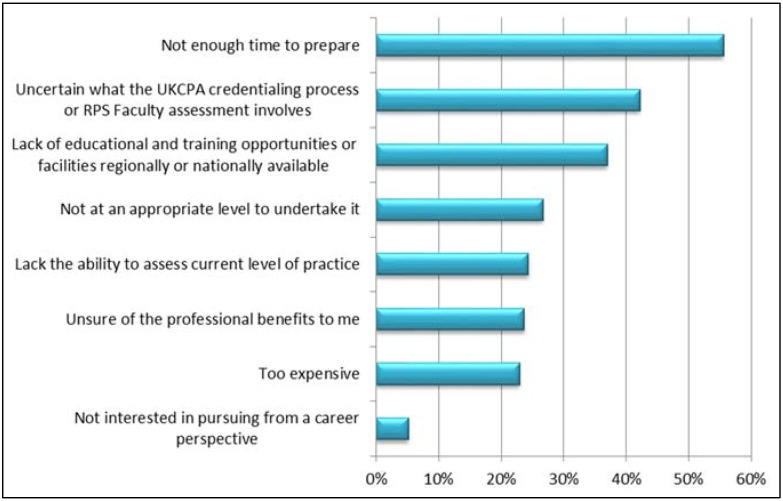
Critical care pharmacist barriers for not undertaking the UKCPA CCG credentialing process or the RPS Faculty accreditation.

Critical care pharmacists identified potential barriers for undertaking the UKCPA credentialing or RPS Faculty accreditation; two-fifths (42.2%, 57/135) were unsure what the process involved and approximately a quarter (23.7%, 32/135) were unsure of its professional benefits (Figure 1). In addition, more than one-third of respondents (37%, 50/135) felt that there was a lack of regional or national education and training opportunties for developing ALP (Figure 1). Interestingly, only 5.2% (7/135) felt that they were not interested in pursuing ALP from a career perspective (Figure 1).

### 3.4. Educational Resources used for Preparation for Advanced Level Practice

**Figure 2:**
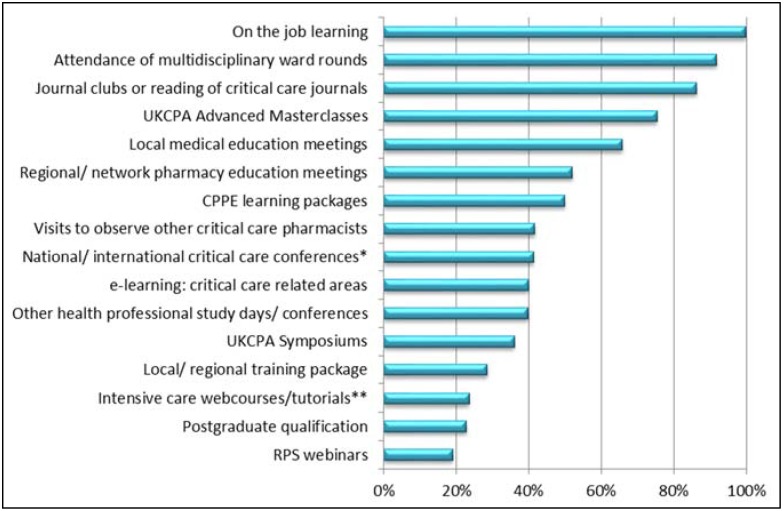
Percentage of critical care pharmacists using particular educational resources to develop their advanced level practice. * European Society of Intensive Care Medicine (ESICM), Intensive Care Society (ICS), International Symposium on Intensive Care and Emergency Medicine (ISICEM); ** ESICM, Association of Anaesthetists of Great Britain and Ireland (AAGBI), Society of Critical Care Medicine (SCCM).

Pharmacists reported using a wide range of educational resources to develop ALP (Figure 2). In particular, they rely on “on the job training”, multidisciplinary ward rounds and journal clubs or reading journals (Figure 2). The UKCPA Advanced Masterclasses were also well accessed by pharmacist responders (76.5%, 98/128) with good perceived benefit (57.1%, 56/98 reported them to be very useful) (Figure 2).

Just over a quarter (28.6%, 36/126) of pharmacist responders reported using a local or regional training package (Figure 2). The majority (80%, 8/10) that then commented, stated they used the Midlands Critical Care Networks (MCCN) Band 7 pharmacist training pack, which is only suitable for developing foundation level practice [9]. Others (20%, 2/10) stated that they used in-house training packages some of which were designed for nurses or anaesthetists.

Pharmacist responders had divided views on whether the current educational resources were adequate for developing ALP. Overall, most disagreed or strongly disagreed (46.7%, 64/137) that the educational resources were adequate, with 40% (55/137) being unsure.

### 3.5. National or Regional Training Programme for Advanced Level Practice

Overall, 89.8% (123/137) of the pharmacist responders agreed (50.4%, 69/137) or strongly agreed (39.4%, 54/137) that there was a need for a national or regional training programme for ALP in critical care. In addition, all the pharmacists who had undertaken the UKCPA credentialing process or RPS faculty accreditation felt this was needed (100%, 20/20; 9/20 agreed and 11/20 strongly agreed, 1 declined to answer). There was no difference between the pharmacists who had been assessed for ALP and the non-assessed group in the proportions supporting the development of a training programme (*p* = 0.217). There was also no difference in the views of pharmacists who worked in a designated teaching hospital or who worked as part of critical care pharmacy team compared to those that did not (*p* = 0.462 and *p* = 1.00, respectively)*.*

### 3.6. UKCPA CCG Masterclasses

The UKCPA CCG currently run two different masterclasses “starting out in critical care” for pharmacists beginning to work in critical care and “advanced practitioner”. The “advanced practitioner” day covers different topics suited to pharmacists working towards or at an advanced level. Approximately four-fifths (78.1%, 107/137) of pharmacist responders had attended at least one UKCPA critical care masterclass with 41.6% (57/137) attending both the “starting out in critical care” and “advanced practitioner” days. Pharmacists identified both benefits and barriers to a critical care conference over the current masterclass system (Table 2).

**Table 2:** Benefits and barriers of a critical care conference over current masterclass study days.

Benefits or Barriers of a Critical Care conference	Frequency n, (%)
Networking, e.g., discussing with peers, sharing best practice and providing advice (Benefit)	110 (84%)
Potential for more topics to be included (Benefit)	103 (79%)
Inclusion of different levels, e.g., beginners, foundation & advanced level (Benefit)	98 (75%)
Potential of a more flexible format (ability to choose sessions of interest to attend) (Benefit)	96 (73%)
Conference fees (Barrier)	92 (70%)
Accommodation costs (Barrier)	75 (57%)
Difficulty obtaining managers permission to attend due to increased number of days (Barrier)	75 (57%)
Difficulty in more than one member of your team being able to attend (Barrier)	69 (53%)
Ward cover would not be provided whilst away (Barrier)	65 (50%)
No barriers to a critical care conference	4 (3%)
No advantages to a critical care conference	2 (2%)

The respondents were asked about their preferences for the format of a critical care conference. The majority (61.2%, 79/129) preferred the option of a mixture of different ability sessions (beginners and advanced level sessions) running simultaneously each day rather than specific days for different levels of practice (e.g., Day 1: Beginner, Day 2: Advanced and Expert). Comments from respondents highlighted that the mixed ability sessions would allow increased networking, learning from peers and experts and tailoring of attendance to their own learning needs. Approximately two-thirds of respondents (66.7%, 86/129) were interested in having a third day for the clinical assessment of the RPS faculty accreditation.

Overall, pharmacists felt that a critical care conference was better for critical care education than the current masterclass days, and 98.5% (129/131) suggested that they would be interested in attending this.

## 4. Discussion

To our knowledge, this is the first questionnaire conducted to obtain the views of UK critical care pharmacists regarding the provision of ALP education. Although the response rate was only 40%, we received representative responses throughout the UK with the views of both UKCPA and non-UKCPA members surveyed. The majority of pharmacists had worked in critical care for more than two years and were Band 8a and above. Hence, they are representative of the national specifications for clinical pharmacist staffing in critical care areas.

The most important finding was that almost all (circa 90%) respondents indicated that they believed a national training package for ALP was required in this pharmacy specialty. This is a clear indication that such a training programme must be developed if the profession is to be able to provide the adequate numbers and level of clinical pharmacists stipulated by the national standards for critical care [5]. Every critical care patient should be reviewed by an advanced level critical care pharmacist; if not, the clinical pharmacist should have access to advice from an advanced level pharmacist. The importance of having input from a senior or consultant-level critical care pharmacist was highlighted by the PROTECTED UK study, which identified that they provided a higher level of care [10].

Currently, the main resources critical care pharmacists used to develop their ALP were “on the job” learning and attendance of multidisciplinary ward rounds. Work-based learning or apprenticeships can be restrictive in their provision of professional development opportunities [11]. In addition, work-based learning has been reported to be insufficient in preparing doctors for the transition to specialist training in a timely manner, under the restrictions of the European working time directives [12]. A reliance on work-based learning by critical care pharmacists may not be an efficient or sufficient way to develop ALP. To accelerate the progression of ALP, critical care pharmacists need more appropriate and targeted educational resources.

The demographics of the respondents suggest that a significant proportion of critical care pharmacists are at a suitable level to undertake the RPS faculty accreditation for Advanced Stage II. Importantly, only a small minority were uninterested in pursuing this from a career perspective. This indicates a large proportion of critical care pharmacists do want to obtain ALP. Interestingly, a significant number of respondents were unsure what the RPS Faculty accreditation process involved and its professional benefits. The RPS may need to address critical care pharmacists’ awareness of the Faculty and its potential benefits. Critical care pharmacists did consider that they were not confident to self-assess their level of clinical practice and identified this as a barrier to undertaking the RPS Faculty accreditation. The literature does support that self-assessment by health professionals is usually poor [13]. In addition, the participants’ feedback from the UKCPA credentialing suggested that undertaking this process increased their confidence in self-assessment but they also highlighted the need for peer review, formal education and guidance [14]. There were divided opinons on whether the current educational resources were adequate for pharmacists to develop their practice to an advanced level. However, a lack of resources was cited as a barrier to undertaking the RPS faculty accreditation. Comments from the respondents suggested that the provision and access to resources may be affected by locality and that pharmacists working in isolation may be at a disadvantage. A national training programme would go some way to address this imbalance. Furthermore, to overcome some of the barriers identified by the pharmacists to undertaking RPS faculty accreditation, any national training programme should incorporate a way for pharmacists to measure their level of practice against the critical care syllabus. Considering the importance of providing the appropriate level of pharmaceutical care to critically unwell patients, the support of the NHS should be sought to help deliver this.

There was minimal use of critical care e-learning packages, web courses or tutorials. The HeXL study has identified many challenges of adopting e-learning amongst NHS healthcare professionals including; its suitability, learner awareness, availability and users learning preferences [15]. However, e-learning has been shown to increase user access and allow more flexible learning [16]. There is great potential for the use of e-learning for critical care pharmacists and it would be important to identify the reasons why it is not being used currently.

Some respondents stated that they use other resources such as British Association for Parenteral and Enteral Nutrition (BAPEN), The European Society for Clinical Nutrition and Metabolism (ESPEN), and The British Society Antimicrobial Chemotherapy (BSAC). This highlights the potential importance for cross over with education and training of other UKCPA clinical specialties. The current UKCPA masterclasses are useful in developing critical care pharmacists’ practice. However, a critical care conference may provide one way to improve the education and training provision for ALP and perhaps complement a national training programme. The critical care pharmacists are supportive of developing a critical care conference.

In summary, the results of this questionnaire have highlighted the inadequate provision of ALP training in UK critical care pharmacy. The NHS should work with current training organisations such as the UKCPA and RPS to ensure this is delivered to support the care of critically ill patients.

### Limitations

Not all critical care pharmacists’ views in the UK have been identified as at least half of the critical care pharmacists in the UK did not respond. In addition, not all of the critical care pharmacists may have received the survey, as we tried to target all of them, but some were contacted through surrogates.

Not all questions were answered by all of the respondents including some of the baseline characteristics. For instance, 6.6% (11/166) did not respond to the question of which country they practice in within the UK. It is possible that some of the pharmacists do not currently work in the UK as the UKCPA website is also used by pharmacists worldwide.

Pharmacists were asked if they worked as part of a critical care pharmacy team. However, we did not explain explicitly what we meant by a “critical care pharmacy team”; for example, this did not allow us to differentiate the different staff skill mix (pharmacists banding, medicine management technicians) and the size of their team.

The questionnaire did not establish the critical care pharmacists’ thoughts on specific gaps in the provision of teaching for certain elements of knowledge or skills related to UKCPA critical care syllabus. Further work should be carried out to identify these gaps to allow more targeted education and training for critical care pharmacists.

## 5. Conclusions

This questionnaire of UK critical care pharmacist opinions on education for developing ALP represented views from the across the UK. The results highlight that currently there are no suitable training packages for ALP and that inefficient work-based learning is the main resource currently used by critical care pharmacists.

To accelerate the progression of critical care pharmacists to ALP in the numbers required nationally, more appropriate and targeted educational resources are required. There is a need for a national or regional training programme to be developed for ALP, and UK critical care pharmacists are supportive of this.

Further research is required to identify an effective education and training model for ALP within critical care. The potential for cross specialty education and training should be explored within this model.

## Footnotes

1 The UKCPA CCG Expert Practice Development Group is comprised of UK pharmacists working at advanced or consultant-level practice in critical care pharmacy who lead the development of pharmacy practice and research in this specialty.

## Appendix A

The Advanced Level Practice education questionnaire;

1. Please indicate your UK country of practice.						
- England - Scotland - Wales - Northern Ireland						
2. How many years of experience do you have working in critical care?						
0–2 years, >2–5 years, >5–10 years, >10 years						
3. Please indicate your posts AFC band:						
6 7 8a 8b 8c 8d 9						
4. Do you work in a designated teaching hospital?						
Yes No						
5. Do you routinely work as part of a critical care pharmacy team? Yes No						
6. Have you previously undertaken either the UKCPA Critical Care Group credentialing process or the Royal Pharmaceutical Society Faculty assessment?						
Yes No						
7. What are the important barriers for not undertaking the UKCPA CCG credentialing or RPS Faculty assessment? Select all that apply;						
						**Select**
Do not believe I am currently at an appropriate level to undertake						
Financial burden excessive						
Lack ability to assess my current level of practice						
Lack of educational and training opportunities/facilities regionally or nationally available for advanced level practice						
Not enough time to prepare for						
Not interested in pursuing from a career perspective						
Uncertain what the UKCPA credentialing process or RPS Faculty assessment involves						
Unsure of the professional benefits to me						
Comments;						
8. What educational resources do you use to develop your advanced level practice? For those that you use, rate how useful you find them. Where 1 is **not** **useful** and 5 is **very useful**						
****	**1**	**2**	**3**	**4**	**5**	**Do Not Use**
Attendance of multidisciplinary ward rounds						
CPPE learning packages						
e-learning—critical care-related areas						
ESICM/ AAGBI/ SCCM webcourses/tutorials (or equivalent), e.g., PACT/learnicu.org						
Journal clubs (e.g., local or UKCPA CCG) or regular review and reading of critical care-related journals						
Local medical education meetings (e.g., Postgraduate Medical Education)						
Local pharmacy educational meetings						
Local/ regional training package (Please state origin & level in the comments box)						
National or international critical care-related conferences, e.g., ICS, ESICM, SCCM, ISICEM meetings						
On the job learning						
Other health professional study days/conferences (Please state organisation in comments box)						
Postgraduate qualification (e.g., MSc in critical care)						
Regional or network-wide pharmacy educational meetings						
Royal Pharmaceutical Society webinars						
UKCPA Advanced Masterclasses						
UKCPA Symposiums						
Visits to other units to observe other critical care pharmacists practice						
Others (Please state in comments box)						
Comments;						
9. The current educational resources available are adequate for developing advanced level practice and for the preparation for the credentialing process.						
Strongly disagree, disagree, neither disagree or agree, agree, strongly agree Comments;						
10. There is a need for National or regional training programmes for critical care advance level practice						
Strongly disagree, disagree, neither disagree or agree, agree, strongly agree Comments;						
11. Have you ever attended a UKCPA critical care masterclass?						
No Yes—“starting out” only Yes—advanced practitioner only Yes—both “starting out” and advanced practitioner						
The UKCPA CCG is considering holding a critical care conference incorporating the material from the masterclass study days delivered over 2 consecutive days with a 3rd day for pre-registered candidates to undergo assessment for RPS Faculty accreditation.						
12. What benefits do you think a critical care conference would provide over the current system of beginner and advanced masterclasses study days? Select all that apply;						
						**Select**
Networking, e.g., discussing with peers, sharing best practice and providing advice						
Potential for more topics to be included						
Potential of a more flexible format (Ability to choose sessions of interest to attend)						
Inclusion of different levels, e.g., beginners, foundation & advanced level						
Other (Please state in comments box)						
None						
Comments;						
13. What barriers may prevent you from attending a critical care conference over the current masterclass study day system? Select all that apply;						
****						**Select**
Conference fees						
Accommodation costs						
Ward cover would not be provided whilst away						
Difficulty obtaining managers permission to attend due to increased number of study days						
Difficulty in more than one member of your team being able to attend						
Other (Please state in comments box)						
None						
Comments;						
14. Two different formats have been suggested for the critical care UKCPA conference. Please select the format you would prefer;						
- Specific conference day designated for different level of practice (e.g., Day 1: Beginner, Day 2: Advanced and Expert) - Mixture of different ability sessions each day (e.g., beginners and advanced level sessions running simultaneously)						
Comments;						
15. Would you be interested in attending a 3rd Day in which you were assessed for RPS Faculty accreditation?						
Yes No						
16. Where do you think the RPS Faculty assessment would be best placed in relation tothe conference?						
- Day before the conference - Day after the conference						
17. What would you be willing to pay for a UKCPA critical care conference fees/ day (if not fully or partially funded by your employer)?						
- £50–100 - >£100–150 - >£150–200 - >£200–250						
18. A UKCPA critical care conference run over 2 days would be a better way to provide critical care education over the current masterclass study days. Strongly disagree, disagree, neither disagree nor agree, agree, strongly agree Comments;						
19. Would you be interested in attending a Critical Care UKCPA conference?						
Yes No						
